# Supplementary material for: The interactome of intact mitochondria by cross-linking mass spectrometry provides evidence for coexisting respiratory supercomplexes
Source: Mol Cell Proteomics. 2017 Dec 8;17(2):216–32. doi: 10.1074/mcp.RA117.000470 (PMC5795388; doi:10.1074/mcp.RA117.000470)
Supplement: Supplemental Data [file supp_RA117.000470_133922_0_supp_23978_fzffwf.zip › spectra_annotation/mito_spectra_annotation/357-1-3-1-6-1.pdf]

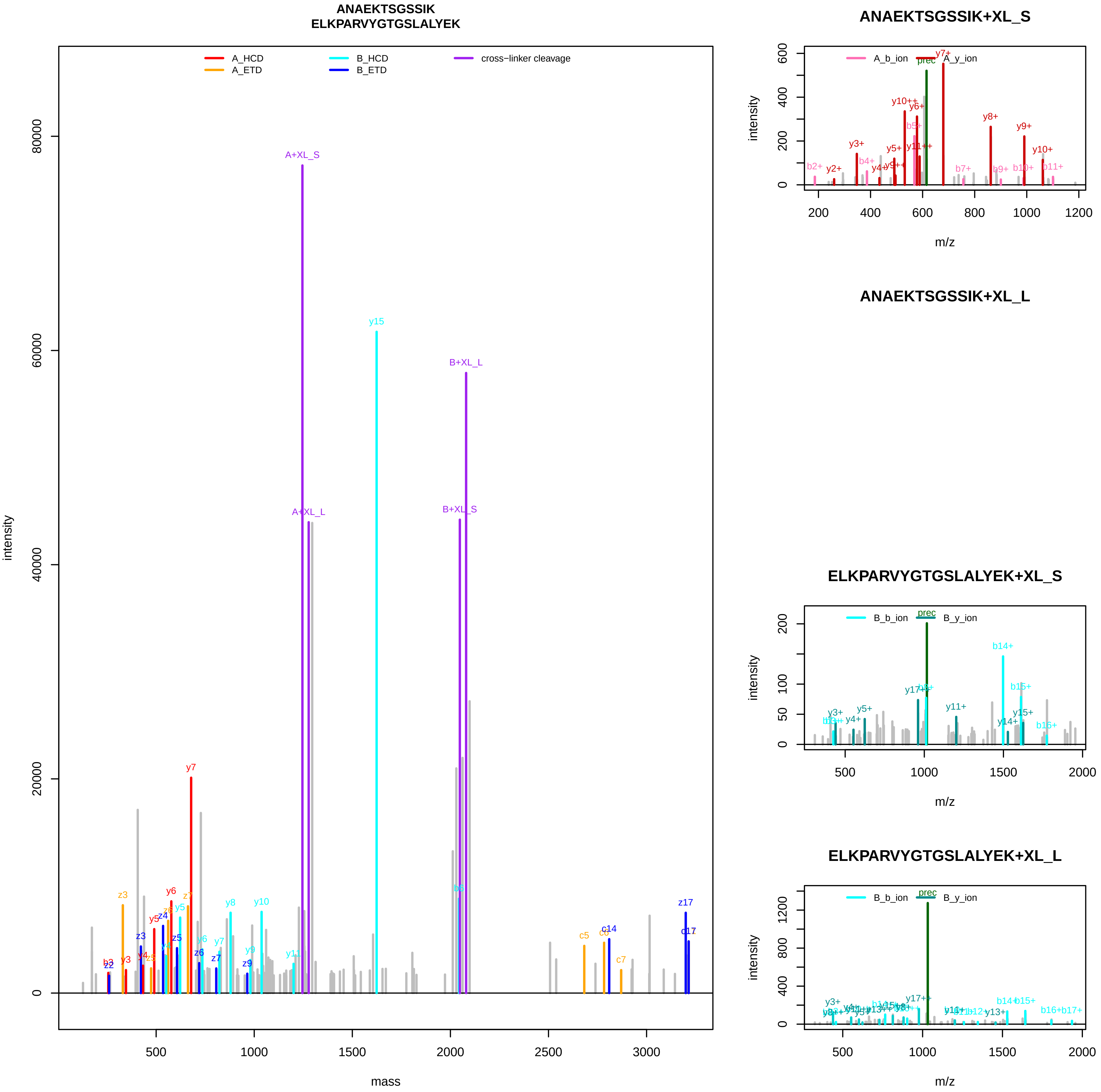

ANAEKTSGSSIK
ELKPARVYGTGSLALYEK
A_HCD
A_ETD
B_HCD
B_ETD
cross−linker cleavage
A+XL_S
A+XL_L
B+XL_L
B+XL_S
y15
y7
y6
y5
z3
z4
z5
z6
z7
z17
c14
c5
c7
c17
y8
y10
y11
intensity
mass
ANAEKTSGSSIK+XL_S
A_b_ion
A_y_ion
prec
m/z
ANAEKTSGSSIK+XL_L
ELKPARVYGTGSLALYEK+XL_S
B_b_ion
B_y_ion
b14+
b15+
b16+
y17+
y11+
y15+
ELKPARVYGTGSLALYEK+XL_L
